# Nest trampling and ground nesting birds: Quantifying temporal and spatial overlap between cattle activity and breeding redshank

**DOI:** 10.1002/ece3.3271

**Published:** 2017-07-28

**Authors:** Elwyn Sharps, Jennifer Smart, Lucy R. Mason, Kate Jones, Martin W. Skov, Angus Garbutt, Jan G. Hiddink

**Affiliations:** ^1^ School of Ocean Sciences Bangor University Menai Bridge Anglesey UK; ^2^ NERC Centre for Ecology & Hydrology Environment Centre Wales Bangor Gwynedd UK; ^3^ RSPB Centre for Conservation Science, RSPB Sandy Bedfordshire UK; ^4^ School of Biological Sciences University of East Anglia Norwich UK; ^5^ Centre for Wildlife Assessment and Conservation School of Biological Sciences University of Reading Reading Berkshire UK

**Keywords:** agri‐environment, animal movements, cow, shorebirds, waders

## Abstract

Conservation grazing for breeding birds needs to balance the positive effects on vegetation structure and negative effects of nest trampling. In the UK, populations of Common redshank *Tringa totanus* breeding on saltmarshes declined by >50% between 1985 and 2011. These declines have been linked to changes in grazing management. The highest breeding densities of redshank on saltmarshes are found in lightly grazed areas. Conservation initiatives have encouraged low‐intensity grazing at <1 cattle/ha, but even these levels of grazing can result in high levels of nest trampling. If livestock distribution is not spatially or temporally homogenous but concentrated where and when redshank breed, rates of nest trampling may be much higher than expected based on livestock density alone. By GPS tracking cattle on saltmarshes and monitoring trampling of dummy nests, this study quantified (i) the spatial and temporal distribution of cattle in relation to the distribution of redshank nesting habitats and (ii) trampling rates of dummy nests. The distribution of livestock was highly variable depending on both time in the season and the saltmarsh under study, with cattle using between 3% and 42% of the saltmarsh extent and spending most their time on higher elevation habitat within 500 m of the sea wall, but moving further onto the saltmarsh as the season progressed. Breeding redshank also nest on these higher elevation zones, and this breeding coincides with the early period of grazing. Probability of nest trampling was correlated to livestock density and was up to six times higher in the areas where redshank breed. This overlap in both space and time of the habitat use of cattle and redshank means that the trampling probability of a nest can be much higher than would be expected based on standard measures of cattle density. *Synthesis and applications*: Because saltmarsh grazing is required to maintain a favorable vegetation structure for redshank breeding, grazing management should aim to keep livestock away from redshank nesting habitat between mid‐April and mid‐July when nests are active, through delaying the onset of grazing or introducing a rotational grazing system.

## 1 INTRODUCTION

Grazing by wild or domestic animals is commonly used to conserve landscapes and ecosystems and to preserve their associated species and communities (WallisDeVries, 1998). Guidelines for conservation management tend to assume that grazing animals distribute themselves homogenously across a landscape (e.g., Adnitt et al., 2007; Green 1986). However, previous studies on the spatial distribution of livestock have found that their distribution can vary markedly in space and depends on numerous biotic and abiotic factors such as the availability of shelter, distance to drinking water, and forage quality and quantity (Bailey, 1995; Putfarken, Dengler, Lehmann, & Härdtle, 2008). These studies have focused mainly on intensively grazed highly managed pasture systems that tend to have a homogenous and species‐poor vegetation with universal accessibility. Few studies have examined the distribution of domestic grazers on botanically and geomorphologically variable habitats with restricted access to some areas, such as saltmarshes.

**Figure 1:**
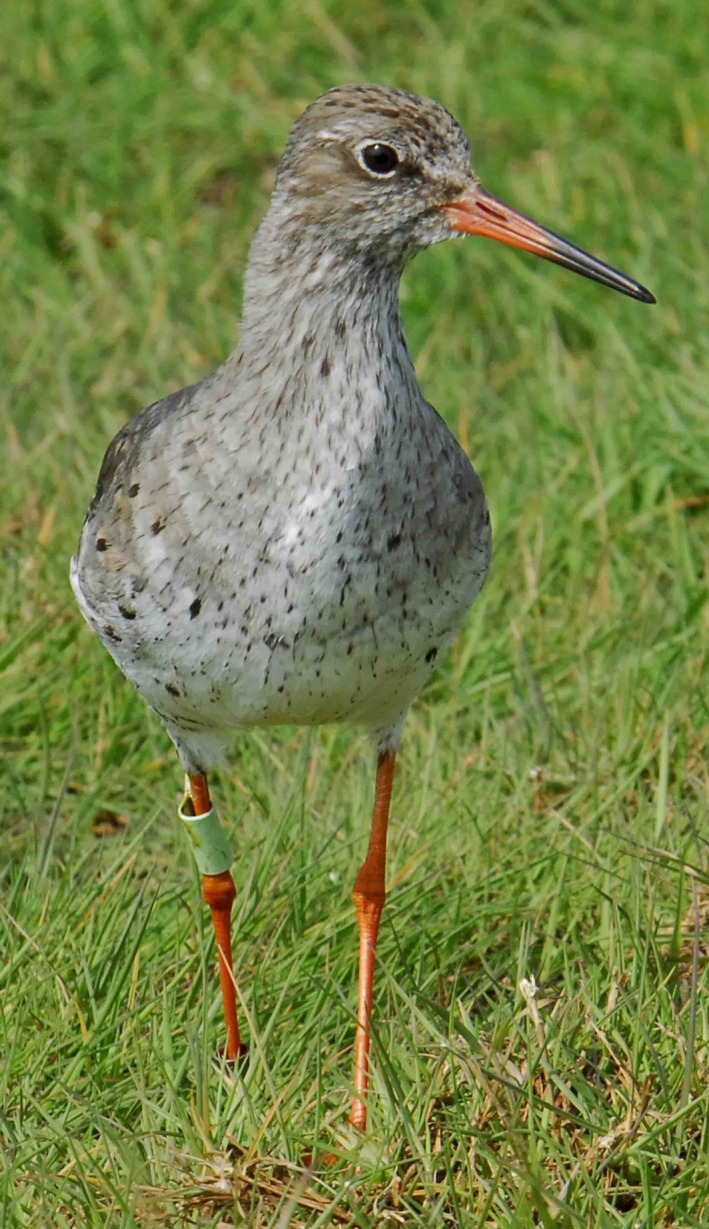
Common redshank *Tringa totanus*. Copyright of Kevin Simmonds

Saltmarshes typically consist of a limited number of plant species adapted to regular immersion by the tides, with a characteristic zonation which ranges from a pioneer zone of extremely halophytic plants adapted to regular tidal immersion at a low elevation, through to a marsh largely composed of grassy less salt‐tolerant species at higher elevations (Boorman, 2003; Gray, 1992). Many saltmarshes are grazed for conservation purposes to optimize sward structure for invertebrates, small mammals, and birds (Boorman, 2003; Davidson et al., 2017). European saltmarshes are an important breeding habitat for a range of ground nesting bird species, for example, common redshank (*Tringa totanus*: hereafter redshank; Figure 1), eurasian oystercatcher (*Haematopus ostralegus*), eurasian skylark (*Alauda arvensis*), and meadow pipit (*Anthus pratensis*). These species tend to nest in the higher elevation saltmarsh zones that are closer to the landward edge and therefore out of reach of most high tides (van Klink et al., 2016; Norris, Cook, Odowd, & Durdin, 1997). On British saltmarshes, numbers of breeding redshank are nationally and internationally important; in the 1980s and 1990s approximately 50% of the British breeding population occurred in this habitat (Brindley et al., 1998). However, redshank breeding on saltmarshes declined by 53% between 1985 and 2011, and this suggests that the current management of saltmarshes is not favorable for redshank (Malpas, Smart, Drewitt, Sharps, & Garbutt, 2013).

Light grazing at an intensity of ~1 cattle/ha can produce the patchy vegetation structure needed for redshank breeding (Norris et al., 1997; Sharps, Garbutt, Hiddink, Smart, & Skov, 2016). Redshank population declines on British saltmarshes have been linked to changes in grazing management as breeding densities are higher in light and moderate grazing than on heavily grazed or un‐grazed saltmarshes (Malpas et al., 2013; Norris et al., 1998). However, Malpas et al. (2013) found that the number of breeding pairs declined by 51.6% in Northern England where grazing was more intensive, but also by 24.2% and 58.1%, respectively, in Eastern and Southern England where light grazing prevailed. The density of animals in a habitat can be a misleading indicator of habitat quality (Van Horne, 1983), as species can preferentially use habitat which acts as an “ecological trap” by lowering breeding success (Best, 1986; Schlaepfer, Runge, & Sherman, 2002). Sharps et al. (2016) demonstrated that grazing creates a trade‐off for Redshank, by causing them to nest in poorer quality habitat but with more of their preferred vegetation types. Even light grazing can reduce redshank nest survival through nest trampling. Sharps, Smart, Skov, Garbutt, and Hiddink (2015) found that risk of redshank nest loss to livestock trampling increased from 16% at 0.15 cattle/ha to 98% at 0.82 cattle/ha on sites in north west England and that nests closer to the landward extent of saltmarshes may be more vulnerable to trampling. In practice livestock tend to be introduced in April or May and remain until September or October to cover the main period of vegetation growth (Doody, 2008). Saltmarsh management guidelines recommend starting grazing in April at an intensity of ~1 cattle/ha (Adnitt et al., 2007), which coincides with the April to July redshank nesting season (Green, 1984). Current conservation grazing management may therefore be causing high rates of nest trampling.

On saltmarshes redshank build nests in the grasses *Festuca rubra*,* Elytrigia* spp., and occasionally *Puccinellia maritima* (Norris et al., 1997; Sharps et al., 2016; Thyen & Exo, 2005), which are found at higher elevations closer to the landward edge of the marsh (Adam, 1990; Allen & Pye, 1992). Grazing pressure can be higher in these areas and lower in the pioneer zone, which is closer to the seaward side of the marsh, possibly because these higher zones are composed of grasses which are more palatable to livestock (Esselink, Fresco, & Dijkema, 2002; Pehrsson, 1988). Livestock density also tends to be higher close to sources of fresh drinking water (Arias & Mader, 2011). On saltmarshes, there are typically no natural sources of freshwater and limited numbers of drinking troughs tend to be placed at the landward side of the marsh (typically 1–3 on a 200–400 ha saltmarsh). When water and food are spatially separated, cattle can spend up to 45% of their time grazing and 25% of their time walking, with the rest of the time spent sleeping or ruminating (Hughes & Reid, 1951).

Diet choice of grazing animals is based on maximizing energy intake and the quality and availability of forage intake (Vulink & Drost, 1991). It is plausible that livestock will first exploit the closest preferred vegetation types and will move onto the less preferred vegetation types further away from drinking troughs as vegetation becomes depleted (van Klink et al., 2016). However, livestock are more likely to forage on previously grazed vegetation as it regrows, rather than on previously ungrazed vegetation (McNaughton, 1984; Nolte, Esselink, Smit, & Bakker, 2014). Therefore, livestock distribution is likely to vary with time, but changes over time may not be linear due to depletion of preferential forage types or the need to return to drinking troughs more often in warm weather. Little is known about how the patchy distribution of livestock in space and time affects nest trampling rates of breeding birds.

The aim of this study was to investigate (i) the spatial and temporal distribution of cattle across the grazing season in relation to the distribution of preferred redshank habitats during the nesting period and (ii) the relationship between nest trampling rates and grazing pressure. Identification of the drivers of the distribution of livestock may allow improvements to grazing management that will maintain positive effects of grazing on the vegetation structure while reducing the negative effects of nest trampling. We hypothesize that: (i) livestock activity is not homogenous over the saltmarsh and is higher in zones where redshank nest; (ii) the furthest distance travelled by livestock increases over the grazing season; (iii) that the probability of nest loss to trampling is higher in parts of saltmarshes where livestock spend more time.

## 2 MATERIALS AND METHODS

**Figure 2:**
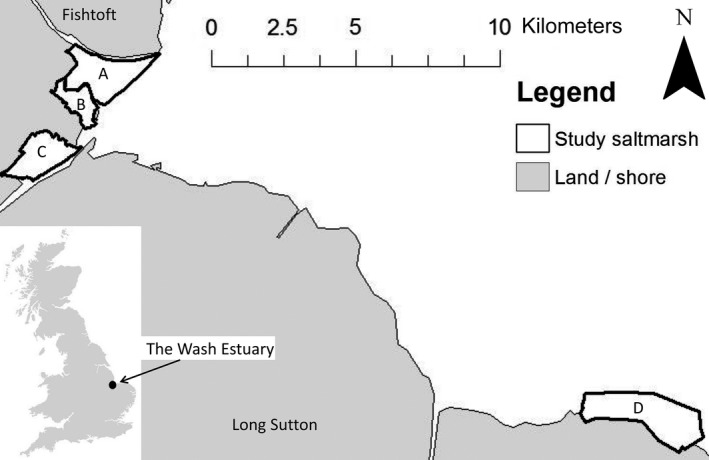
Wash estuary, showing the study saltmarshes. A and B: Frampton Marsh, C: Kirton Marsh, D: Terrington Marsh. Although saltmarshes A and B are neighboring, they are separated by a large channel which is unpassable to livestock. Close to the landward edge of the marsh where the channel narrows, fencing has been installed. This means that livestock are unable to move between the two saltmarshes

This study was carried out on four saltmarshes of the Wash estuary with grazing intensities well below the recommended ~1 cattle/ha (0.11–0.50 cattle/ha; Table 1; Figure 2). To investigate drivers of the spatial and temporal variation in livestock distribution, we used GPS loggers placed on cattle. To relate cattle density to avian nest loss due to trampling, we used dummy nests.

**Table 1:** Saltmarshes used in this study, showing seasonal cattle density per hectare (SCD) and GPS logger details

Salt‐marsh	Size (ha)	Herd size	SCD ha^−1^	LSU ha^−1^	No. cattle GPS tagged	Dates GPS logged	No. GPS positions	No. of cattle days
A	322	116	0.36	0.29	4	19/05/13–10/08/13	11,819	205
B	126	39	0.31	0.25	4	19/05/13–26/10/13	31,958	432
C	201	100	0.50	0.40	5	28/04/14–20/07/14	23,967	326
D	477	60	0.13	0.10	3	05/05/14–17/08/14	11,328	105

LSU = livestock units. Cattle days are the number of days of cattle activity recorded from active collars.

### 2.1 Field sites

The Wash estuary contains over 4,000 ha of saltmarsh, which is approximately 10% of the total UK saltmarsh extent (Burd, 1989; Murby, 1997). The vegetation is typical of saltmarshes on the east coast of the UK. *Salicornia* and other annual plant species form pioneer communities along with *Spartina anglica* at the lowest elevations. The mid‐marsh areas are dominated by *Puccinellia maritima* communities, which form a short turf with occasional tussocks across most of their extent where grazed by livestock. In areas where livestock activity is limited or absent, the low growing shrub *Atriplex portulacoides* and the coarse grass *Elytrigia atherica* dominate, mainly through the central and upper parts of the marsh extending on to the vegetated flood defenses (Hill, 1988; Murby, 1997). All saltmarshes included in this study were bounded by a vegetated sea‐wall flood defense at the landward edge. The study saltmarshes were grazed by free‐roaming young cattle, which is commonplace on British saltmarshes (Adnitt et al., 2007). Young cattle may trample more nests than adults (Beintema & Muskens, 1987) possibly due to their more lively nature (Ausden, 2007).

Redshank populations have declined in the Wash estuary (Malpas et al., 2013). At Saltmarshes A, B, and C redshank populations decreased from approximately 140 pairs/km^2^ to around 50 pairs/km^2^ despite maintaining light grazing regimes between 0.3 and 0.6 cattle/ha (Feather, Mason, Smart, & York, 2016). Trends are not known for Saltmarsh D, but the site currently maintains a breeding redshank population of approximately 30 pairs/km^2^ (Jones, 2014).

### 2.2 GPS tracking

Eight cattle were fitted with GPS loggers on saltmarshes A and B from May to October 2013, and eight cattle were fitted with GPS loggers between April and August 2014 on saltmarshes C and D (Table 1). Although this number only represents 3%–10% of the animals in each herd, as cattle are herding animals (Howery, Provenza, Banner, & Scott, 1996, 1998), we assumed that the distribution of this subsample would be representative of the whole herd. GPS loggers were programmed to log a position every 20 min, when satellite signals were available. They were retrieved at the end of the grazing season. Although some collars stopped earlier than planned due to battery life, approximately 50% of the collars per saltmarsh logged the entire period. The logging dates, number of GPS positions, and number of cattle days for each of the saltmarshes are shown in Table 1.

Arc‐GIS 10.1 was used to produce a 50 × 50 m grid over each saltmarsh, and to count the number of GPS records that fell into each grid cell per week. To obtain estimates of livestock density per cell, firstly the area of saltmarsh per grid cell was calculated by subtracting the area of any creeks and any area which fell outside of the saltmarsh boundary. Due to the accuracy of the GPS chipsets (recorded accuracy = 2.5 m), only grid cells which contained saltmarsh >6.25 m^2^ were included in the analyses. Cattle activity was calculated as cattle hours ha^−1^ hr^−1^, which simplifies to cattle/ha, and therefore took account of both the number of cattle and the duration of their presence in a cell. This measure represents the average cattle abundance in a cell over the evaluated time period and was calculated using the formula:

$$\begin{array}{cc} \text{Cattle activity}\;({\text{ha}}^{-1})=\; & \text{Herd size}\times (\text{No. GPS positions in cell}/\\ & \text{Total No. GPS positions})/\text{Cell area}\;(\text{ha}). \end{array}$$

### 2.3 Distribution of cattle activity and distance travelled

To quantify changes in cattle distribution over time, we calculated the percentage of grid cells that contained 100% of the cattle activity for each week (CA_100_). If CA_100_ is large, cattle use a larger fraction of the saltmarsh, and therefore, their activity is more spread out. We used a generalized least squares model (GLS) in the nlme package in the statistical program R (Pinheiro, Bates, DebRoy, & Sarkar, 2016), to test how CA_100_ was affected by saltmarsh identity (A–D) and time (weeks, a continuous variable with week 1 starting on the 14th April as the start of the redshank nesting season). The response variable was log_10_ transformed to deal with uneven spread in the residuals. A quadratic term for time (week^2^) and an interaction between saltmarsh and week (and saltmarsh and week^2^) were also included in the global model. To account for temporal autocorrelation, an auto‐regressive model of order 1 was run, by adding the correlation structure term (corAR1, form = ~week|saltmarsh). The form argument specified the temporal order of the data (the variable “week”). By adding the grouping variable “saltmarsh,” the correlation structure was only applied to observations within each saltmarsh. In this, and all subsequent analyses model selection was carried out by removing single terms from the global model until only predictors with *p* < .05 remained.

To investigate seasonal trends in livestock use of different saltmarsh habitats, we mapped the zonation of each saltmarsh in a field survey and then validated these maps using aerial photographs to create a GIS layer of zonation for each saltmarsh (Figs S1–S4), based on the suitability for redshank nesting. The saltmarsh zones that redshank use for nesting were easily recognizable as they select nests surrounded by grasses such as *F. rubra*,* P. maritima*, or *Elytrigia* species (Norris et al., 1997; Sharps et al., 2016; Thyen & Exo, 2005). The categories used (listed in order of proximity to the sea wall) were as follows: non‐saltmarsh zone (the transition zone between saltmarsh and terrestrial vegetation, and any other nonsaltmarsh areas which the cattle could access), mid‐marsh redshank zone (dominated by *P. maritima* or *F. rubra* and found at high/mid elevation), *Elytrigia* redshank zone (dominated by *E. atherica* and found at high/mid elevation), non‐redshank zone (dominated by *Atriplex* and/or pioneer vegetation, and found at low elevation). We then identified the areas of each grid cell that fell within each of the habitat categories. Where a grid cell fell within more than one habitat zone, we used the habitat zone that occupied the largest area of the grid cell.

A general linear model (GLM, with Gaussian error) of the effect of saltmarsh identity and time (weeks) on cattle activity in each zone was fitted separately. A quadratic term for time (week^2^) and an interaction between saltmarsh and time (and saltmarsh and week^2^) were also included because an initial examination of the data indicated a humped‐shaped relationship between cattle density and time. Where necessary, the response variable was transformed (square root or log_10_ + 1) to ensure normality of residuals and deal with heteroscedasticity. Following Zuur, Ieno, Walker, Saveliev, and Smith (2009) data were tested for temporal autocorrelation by running the global model for each habitat zone, using generalized least squares and inspecting autocorrelation function plots. There was no evidence of temporal autocorrelation.

To determine whether the maximum distance livestock travel from the sea wall varies with time, for all grid cells visited by livestock, the GLS model set was repeated, using the 95th percentile of the distance of all GPS records from the sea wall as the response variable. The 95th percentile was used to exclude any extreme outliers, for example, one off trips to a distant point. We did not use a 5th and 50th percentile as our focus was the maximum distance travelled.

### 2.4 Nest loss to trampling

To allow greater replication than would be possible studying redshank nests, to determine whether the probability of nest loss to trampling is higher in parts of saltmarshes where livestock spend more time, we ran a dummy nest experiment using 110 mm black clay‐pigeon shooting targets which have a similar diameter to redshank nests (e.g., 4 redshank eggs approximately 45–48 mm per egg), and like eggs they break if stepped on by livestock (Jensen, Rollins, & Gillen, 1990; Mandema, Tinbergen, Ens, & Bakker, 2013). This experiment could only be carried out on one of the four saltmarshes, but we expect the relationship between cattle density and trampling rate to be similar across study sites. Thirty positions were selected using a stratified random sampling method across Saltmarsh B, to cover the full range of distances from the sea wall, and all habitat zones (minimum distance between points = 50 m). At each of the 30 plots, nine discs were placed in grids of 9 m × 9 m, with 3 m between each disc. As preliminary observations suggested that cattle behavior was not affected by the presence of the black disks, we laid them directly onto the marsh without cover. The precise location of each disc was recorded using a Leica Viva GS08 Global Navigation Satellite System (accuracy 60 mm; Fig. S2). Discs were exposed to cattle on 22/5/13 when the cattle were first introduced to the saltmarsh during the mid‐April to mid‐July redshank nesting season (Green, 1984). They were checked after 14 days (5/6/13—period 1) and 28 days (19/6/13—period 2). Disks were recorded as intact (not trampled) or broken (trampled). All discs were recovered. When checking discs after period 1, broken discs were replaced with a new disc and all debris was removed. When checking discs after period 2, all intact discs and debris were removed. The daily trampling probability for both 14 day periods was calculated as:

$$\text{Daily trampling probability}=1-{\left(1-\text{trampling.prob.period}\right)}^{1/14}.$$

However, as the incubation period is 24 days for redshank and similar for many other shorebird species (Green, 1984), trampling probability (%) over 24 days was calculated based on the mean of the daily trampling probabilities of the two periods as:

$$\text{Trampling probability for 24 days}=1-{\left(1-\text{daily trampling probability}\right)}^{24}$$

It is expected that the relationship between the probability of nest trampling and cattle activity reaches an asymptote at high cattle densities. Trampling probability was therefore compared to cattle activity, for the 24‐day period using a binomial Generalized Additive Model (GAM) to fit this relationship using R. The data were tested for spatial autocorrelation following Zuur et al. (2009) and Kubetzki and Garthe (2007), this indicated that independence could be assumed (Zuur et al., 2009); therefore, the final model used was a GAM with a smoothed term for cattle density and no additional terms to allow for spatial autocorrelation. Trampling probability maps were created for each saltmarsh by scaling cattle activity recorded over the first 24 days of grazing to model predictions from the GAM.

## 3 RESULTS

### 3.1 Distribution of cattle activity

**Figure 3:**
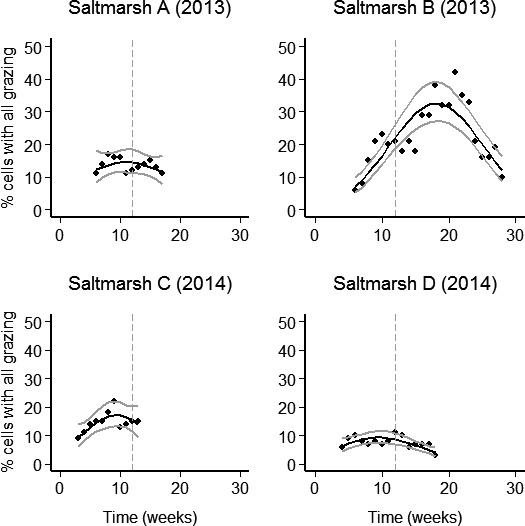
Changes in the percentage of saltmarsh that was grazed over time. The percentage of cells containing all of the grazing is used as a measure of homogeneity of livestock distribution. Black lines are back‐transformed model‐fitted values. Confidence intervals (95%) are indicated by gray lines. The dashed gray vertical lines indicate the end of the redshank nesting season (1st July). Week 1 was the first week of the redshank season, beginning 14th April. Week 28 (the last week) ended on the 26th October

**Figure 4:**
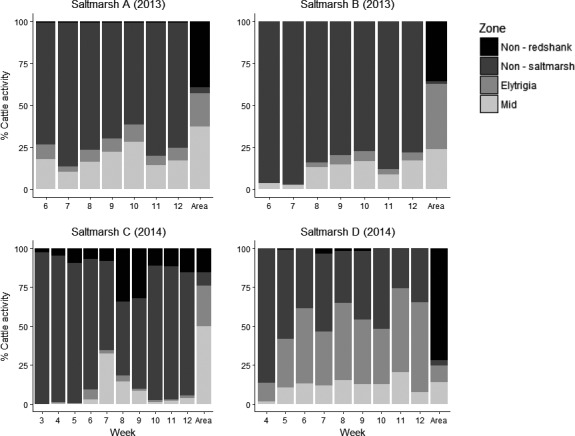
The percentage of cattle activity in the different habitat zones during the redshank nesting season. Week 1 was the week beginning 14th April. Week 12 ended on the 7th July. In Saltmarsh A and B grazing started in Week 6 (19th April), In Saltmarsh C, grazing started in Week 3 (28th April). In Saltmarsh D, grazing started in Week 4 (5th April). The “Area” category on the *X*‐Axis indicates the proportion of each habitat zone present on the saltmarsh in question. Redshank breed in the *Elytrigia* and Mid zones. The non‐redshank and non‐saltmarsh zones are unsuitable for Redshank breeding

The spatial extent of cattle activity was highly skewed, and varied by saltmarsh (Table 2) with between 58% and 78% of the saltmarsh never visited by cattle during the study (Figure 3). Cattle activity varied by habitat zone (Table 3) with most activity concentrated on the habitat zones close to the seawall, in non‐saltmarsh habitat and in redshank nesting areas (Figure 4). Over time, cattle activity moved away from the non‐saltmarsh habitat. In the mid‐marsh redshank habitat, cattle activity gradually increased over the course of the redshank nesting season, but then decreased after the redshank nesting season had finished (Figs S5–S8). The spatial extent of livestock activity increased over time and then decreased again, but the timing of the maximum spread of cattle activity was different between the four saltmarshes. In Saltmarsh B, this maximum spread occurred in August (week 19) with 42% of the available marsh, and in Saltmarsh C, this occurred in June (week 9) with 22% of the available marsh. In Saltmarshes A and D, cattle never used more than 17% of the available marsh (Figure 3).

**Table 2:** Results of general linear models and generalized least squares models investigating spatial and temporal effects on livestock distribution and livestock activity (CA_100_ = % of grid cells with 100% of the cattle activity)

Response variable	Predictor	*df*	Res *df*	*F*	*p* value
CA_100_	Saltmarsh (A‐D)	3	49	22.99	<.001
	Week	1	49	18.24	<.01
	Week^2^	1	49	10.88	<.01
	Saltmarsh*week	3	49	15.35	<.001
	Saltmarsh*week^2^	3	49	0.20	.89
95th percentile of distance to sea wall	Saltmarsh (A‐D)	3	49	5.90	<.01
	Week	1	49	107.81	<.001
	Week^2^	1	49	1.68	.20
	Saltmarsh*week	3	49	11.73	<.001
	Saltmarsh*week^2^	3	49	2.88	.04

**Table 3:** Results of general linear models investigating variation in livestock distribution in different saltmarsh zones over time

Response variable	Predictor	*df*	*F*	*p* value
Cattle activity (ha^−1^) in the non‐saltmarsh zone	Saltmarsh (A‐D)	3, 49	7.1	<.001
	Week	1, 49	5.7	.02
	Week^2^	1, 49	0.5	.48
Cattle activity (ha^−1^) in mid marsh redshank zone	Saltmarsh (A‐D)	3, 49	15.7	<.001
	Week	1, 49	1.9	.17
	Week^2^	1, 49	6.6	.01
Cattle activity (ha^−1^) in *Elytrigia* redshank zone	Saltmarsh (A‐D)	2, 41	65.2	<.001
	Week	1, 41	2.5	.12
	Week^2^	1, 41	0.0	.93
Cattle activity (ha^−1^) in non‐redshank zone	Saltmarsh (A‐D)	2, 29	45.7	<.001
	Week	1, 29	1.7	.20
	Week^2^	1, 29	5.1	.03

*df* = degrees of freedom. Res *df* = Residual degrees of freedom. *F* = F value. For each response variable, we included saltmarsh*week, and saltmarsh*week^2^ in the model, but these were not significant.

### 3.2 Furthest distance travelled

**Figure 5:**
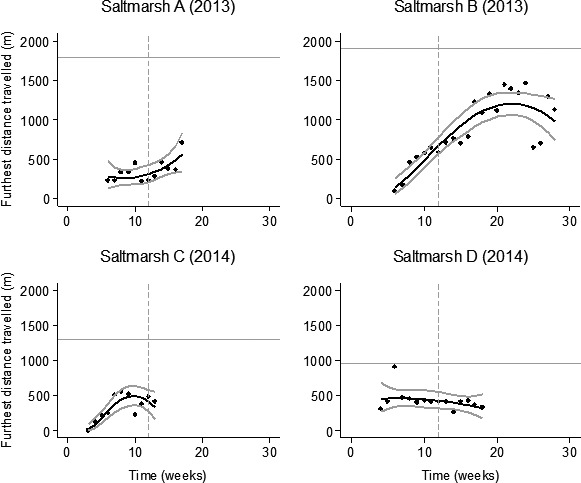
95th percentile of livestock distance to sea wall over time. Black lines are back‐transformed model‐fitted values. Confidence intervals (95%) are indicated by gray lines. The straight horizontal gray line indicates the maximum extent of the saltmarsh in meters. The dashed gray vertical lines indicate the end of the redshank nesting season (1st July). Week 1 was the week beginning 14th April. Week 28 (the last week) ended on the 26th October

At the start of the redshank breeding season most livestock stayed within 500 m of the seawall, but were recorded further afield on some saltmarshes over time as suggested by the 95th percentile of the distance of all GPS records from the sea wall (hereafter referred to as furthest distance travelled; Table 2; Figure 5). At Saltmarsh B, where cattle activity was recorded for the longest period, the furthest distance travelled increased from 129 m in May (week 6) to 1,500 m in September (week 22), but decreased to 1,189 m in October (week 26). This pattern of furthest distance travelled by livestock increasing over time was quadratic. As the effect of the interaction between saltmarsh and time on the maximum distance travelled by livestock was significant, the timing of the maximum travel varied between the saltmarshes. This can be expected as the stocking density, size, and vegetation were different between the different saltmarshes.

### 3.3 Nest loss to trampling

**Figure 6:**
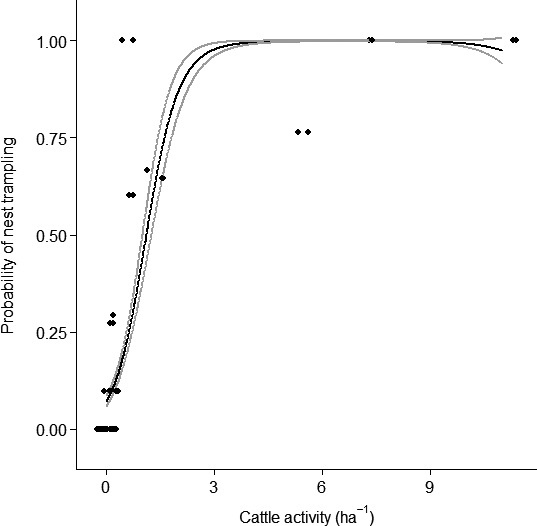
The probability of nest loss to trampling in relation cattle activity (ha^−1^). Black points indicate the study plots (false nests), and these have been jittered to display overlapping data points side by side. The black line is the model predicted values from the GAM. Gray lines indicate 95% confidence intervals

**Figure 7:**
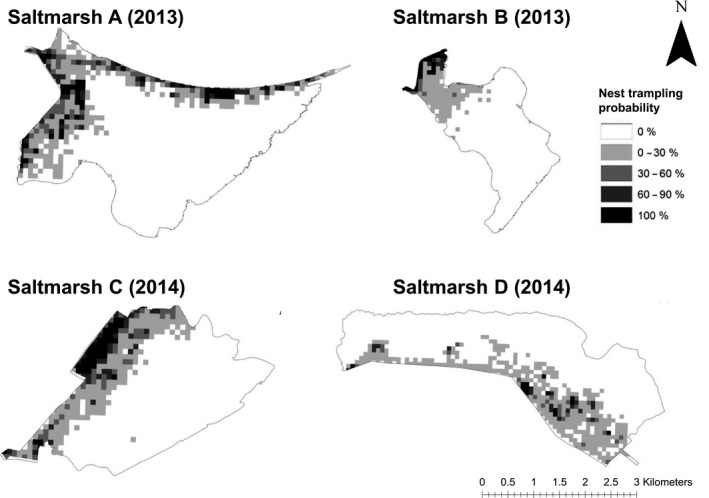
The probability of nest loss to trampling on saltmarshes A–D. Calculated using model fitted values from Figure 6. See Figs S1–S4 for habitat maps of each saltmarsh

The experimental plot that received the most grazing during the false nest experiment recorded cattle density of 11.29 cattle/ha, which is around 36 times higher than mean seasonal cattle density at this saltmarsh (B: 0.31 cattle/ha). The probability of nest trampling over a 24‐day period increased from zero where no cattle were recorded to 100% with cattle >3 ha (Figure 6, *R* ^2^ = 0.75, edf = 1.99, Ref. *df* = 2, χ^2^ = 452.1, *p* < .001 for smoothed cattle density term). Figure 7 presents the nest trampling probability recorded for each of the saltmarshes. This demonstrates that nest trampling rates are highly concentrated at some parts of the saltmarshes, particularly in areas close to the sea wall.

## 4 DISCUSSION

These results show that cattle distribution on coastal saltmarshes is highly concentrated, with only 3%–42% of each saltmarsh being grazed, with much spatial and temporal variation. Early in the grazing season cattle concentrate on higher elevation habitats close to the sea wall, and move out further onto the saltmarsh as the season progresses. As redshank also nest in these higher elevation habitats, and breeding coincides with the early period of grazing (Adam, 1990; Hale, 1980), this pattern of grazing causes a much higher nest loss to trampling than would be expected merely based on the mean density of cattle on the saltmarsh, and means that some parts of the saltmarshes are grazed much more heavily than may be intended while large areas go completely ungrazed. This overlap in the habitat use of cattle and redshank means that the trampling probability of nests can be very high.

Livestock grazing is used as a management tool for conserving numerous target species and communities in a wide range of landscapes and ecosystems (WallisDeVries, 1998), including heathlands, grasslands, and woodlands (Bakker, De Bie, Dallinga, Tjaden, & De Vries, 1983; Eglington et al., 2009; Smart, Gill, Sutherland, & Watkinson, 2006). It may be expected that nest trampling pressure for ground nesting birds would be less in habitats with a uniform coverage of vegetation types preferred by livestock, and multiple sources of drinking water. On saltmarshes, livestock movements are also likely to be influenced by tidal conditions and the weather, which can be more extreme than terrestrial habitats due to their exposed locations (Yasué, Quinn, & Cresswell, 2003). By definition, saltmarshes are affected by varying degrees of tidal flooding (Adam, 1990). Total immersion of saltmarshes by sea water can occur on the highest tides of the spring neap tidal cycle (Armstrong, Wright, Lythe, & Gaynard, 1985), when livestock are forced to retreat to areas with high elevation such as the sea wall (Jensen, 1985). This may suggest that rates of nest trampling are higher on saltmarshes than in terrestrial habitats and highlights a need to change conservation management practices for redshank breeding on saltmarshes.

Because even light grazing of saltmarshes can lead to high rates of nest loss to trampling and predation (Sharps et al., 2015) and causes a trade‐off for redshank by increasing the availability of suitable nesting habitat, but reducing its quality (Sharps et al., 2016) it is likely that this is trade‐off is causing an ecological trap for redshank and contributing to the redshank population declines found by Malpas et al. (2013). Previously grazed saltmarsh vegetation is more palatable to cattle and therefore more likely to be re‐visited (Bakker, 1985). Therefore, if light grazing occurs over a number of years, cattle are likely to select the same preferred areas. As our study shows that cattle only ever use a small proportion of the saltmarsh, we expect that over time an increasing proportion of a lightly grazed saltmarsh is never visited by cattle and therefore becomes less suitable for redshank. This would likely force more redshank into the cattle preferred areas bringing them more and more into conflict. This suggests that there is a need for habitat managers to focus on balancing the trade‐off between improving the quality of the habitat by reducing nest trampling and predation rates (Sharps et al., 2015, 2016), while keeping the positive effects that grazing has of increasing the availability of preferred grass species (Sharps et al., 2016).

As we found that the probability of nest loss to trampling was higher in areas of saltmarshes subject to more livestock activity, our results show that GPS tagging from 3% to 10% of cattle in a herd can be a good indicator of nest trampling probability. As we used false nests to calculate nest trampling probability, and they were placed following a stratified random sampling method, we were concerned that this may bias our findings as redshank do not select nest locations at random (Sharps et al., 2016). It is also unclear if cattle footfall is random, although previous studies suggest that they trample birds' nests in either long or short vegetation, and do not avoid grassy tufts where redshank nest (Beintema & Muskens, 1987; Pakanen, Luukkonen, & Koivula, 2011; Sharps et al., 2015). Although it would have been useful to also study real redshank nests, this would have been time‐consuming and therefore not possible alongside the current study. However, Sharps et al. (2015) studied real redshank nests and found higher rates of nest trampling near the sea wall, on lightly grazed saltmarshes with high livestock densities. As our results demonstrate that livestock activity is largely concentrated in these areas, it is unlikely that using false nests affected our conclusions. Our preliminary observations suggested that cattle behavior was unaffected by the presence of the false nests. If cattle had avoided the false nests, this would underestimate trampling meaning our already high estimates are conservative.

These results demonstrate that understanding the mechanisms driving the spatial habitat use of cattle is important when formulating management strategies for ground nesting birds. In our study, livestock distribution and the maximum distance travelled by livestock increased with time and then decreased again. This could be related to simple food depletion on the higher elevation saltmarsh zones, if cattle are forced to venture further afield once vegetation closer to the sea wall has been heavily grazed, or during periods of slow vegetation growth. This trend appeared to reverse later in the summer months after the redshank breeding season perhaps as temperatures became too high for cattle to move far away from drinking water or as vegetation closer to the sea wall recovered from early season grazing. This has previously been demonstrated in North American pasture systems, where cattle stay close to their drinking water during the hottest periods (Bailey, 1995). The fact that livestock remained close to the sea wall for the majority of the grazing period could either be because this is where fresh drinking water sources are provided, or because vegetation in higher elevation zones in more palatable to livestock (Pehrsson, 1988). The sea wall is often where livestock are first introduced to the saltmarsh and represents a safe dry area during high tides (Doody, 2008). Livestock may therefore associate it with safety which might explain lack of movement from this area in the early part of the grazing period. Higher elevation habitats closer to the sea wall are also drier and less muddy as high tides seldom over‐top these areas and dense vegetation growth consolidates sediments (Adam, 1990), so may be preferred through allowing easier livestock movement. This could explain the higher rates of nest trampling found in some dummy nests during our study.

While these results show a high concentration of livestock activity on parts of the saltmarsh that are most important for breeding redshank and several other bird species, the highest levels of livestock activity were found in the non‐saltmarsh habitats closer to the landward extent of the saltmarsh, and this effectively draws cattle away from the breeding habitats. Such access to non‐saltmarsh habitat is absent at many grazed saltmarshes (Skelcher, 2010). At these locations, it is likely that nest loss to trampling would be even greater as livestock activity may be further concentrated in the mid marsh.

### 4.1 Synthesis and applications

The results of this work do not suggest that stopping livestock grazing on saltmarshes altogether will result in increased nesting success or breeding populations of redshank, because grazing also causes changes in vegetation structure that are beneficial to redshank, by opening the vegetation sward increasing the availability of patchy vegetation that is used for redshank nesting (van Klink et al., 2016; Sharps et al., 2016). Grazing is therefore an important part of saltmarsh management (Brindley et al., 1998; Norris et al., 1997, 1998). Cessation of grazing in previously grazed saltmarshes can result in reductions in numbers of breeding redshank as the vegetation becomes dominated by tall uniform vegetation which is unsuitable for redshank nesting (Norris et al., 1997). Furthermore, livestock grazing of saltmarshes can drive abundance and diversity of invertebrate prey (Ford et al. 2013). If UK Environment Agency guidelines are followed, grazed saltmarshes would have livestock present from April until October (Adnitt et al., 2007).

Several management measures could be considered to reduce the strength of the trade‐off between grazing to maintain a suitable vegetation structure with the need to minimize nest trampling:

1. As our results show that cattle did not move more than 500 m away from the seawall in three of four marshes, grazing densities could be calculated only over the area of saltmarsh within 500 m of the sea wall then scaled to fulfill the 1 cattle/ha grazing recommendation (Norris et al., 1997). This approach would mean that the grazing intensity is adjusted to account for the higher livestock distributions close to sea wall in the most sensitive part of the saltmarsh for redshank. However, the exact distance from the seawall will have to vary for individual saltmarshes depending on the size of the redshank nesting zone, which may render this method impractical due to time constraints of land managers.
2. An alternative approach would be to delay the start date of grazing. Livestock are generally introduced in April or May because this is when vegetation starts to grow (Adnitt et al., 2007); therefore, bringing the start of grazing forward is not feasible. However, as the redshank nesting season lasts from mid‐April to mid‐July, grazers could be introduced when the redshank breeding season has finished. In other habitats, such as lowland wet grasslands, commencing grazing after the end of July has been shown to increase productivity in redshank and other shorebirds (Green 1986). The cattle stocking density would probably need to be higher overall to graze down the vegetation that has built up and to prepare the vegetation for the next spring. This would completely eliminate trampling of nests and might maintain the desired vegetation structure through grazing, although graziers would need to find alternative pasture early in the season. As breeding redshank are highly site faithful, but respond to changing vegetation conditions (Sharps et al., 2016; Thompson & Hale, 1989), this option may be preferable.
3. Alternatively, a rotational grazing regime where saltmarshes are grazed heavily in 1 year and left ungrazed in alternate years may improve breeding success by eliminating nest trampling in the ungrazed year. The saturating nature of the response of trampling probability to livestock grazing suggests that although this approach is likely to lead to total nest loss in the grazed year, it will reduce average nest loss over 2 or more years. Rotational grazing could be carried out using whole marshes or within smaller sections within marshes. This could require some fencing, which can be expensive and impractical in tidal areas where fences may accumulate debris, but creeks could be used as barriers to ensure lengths of fences are shorter. Compartments would need to enable access to water troughs and high tide refuges, which most likely would mean incorporating a section of seawall. However, care would need to be taken with this approach to ensure breeding redshank are not actively selecting the compartments with active grazing. This approach will only work if grazing in alternate years would keep the sward in a suitable condition for nesting.
4. Fencing off redshank habitat completely in the breeding season may be possible but is unlikely to be feasible as a routine solution as the grazers will need access to refuges from flooding during spring tides.
5. The strategic placement of water troughs further away from breeding areas could naturally restrict livestock movements. This approach is unlikely to be effective on a saltmarsh, as water troughs need to be located close to the landward extent of the marsh allow water to be piped to the trough, and so that cattle can access fresh water even during high tides.
6. Finally grazers other than cattle could be considered, but are unlikely to solve the problem. Sheep are more likely to produce shorter vegetation swards, which is unsuitable for redshank (Green 1986; Beintema & Muskens, 1987) and horses cause even higher trampling of nests (Mandema et al., 2013).

In conclusion, this work shows that the areas of the saltmarsh where redshank breed are much more intensively grazed during the breeding season than is desirable, because livestock concentrate in these areas. This results in high nest trampling probability; therefore, changes in grazing management on saltmarshes are necessary to increase the nesting success of redshank. Grazing management should aim to keep livestock away from redshank nesting habitat between mid‐April and mid‐July through delaying the onset of grazing or introducing a rotational grazing system. Trial management is required to test which of these options would maintain a favorable vegetation structure for redshank breeding, while reducing redshank nest loss.

## CONFLICT OF INTEREST

None declared.

## AUTHORS' CONTRIBUTIONS

E.A.S., J.S., L.R.M., M.S., A.G., and J.G.H. conceived the ideas and designed methodology; E.A.S. analyzed the data and led the writing of the manuscript; K.J. collected data in year 2 of this study. All authors contributed critically to the drafts and gave final approval for publication.
