# Supplementary material for: Nest trampling and ground nesting birds: Quantifying temporal and spatial overlap between cattle activity and breeding redshank
Source: Ecol Evol. 2017 Jul 28;7(16):6622–33. doi: 10.1002/ece3.3271 (PMC5574750; doi:10.1002/ece3.3271)
Supplement: Supplementary file 1 [file ECE3-7-6622-s001.docx]

Supplementary Online Material


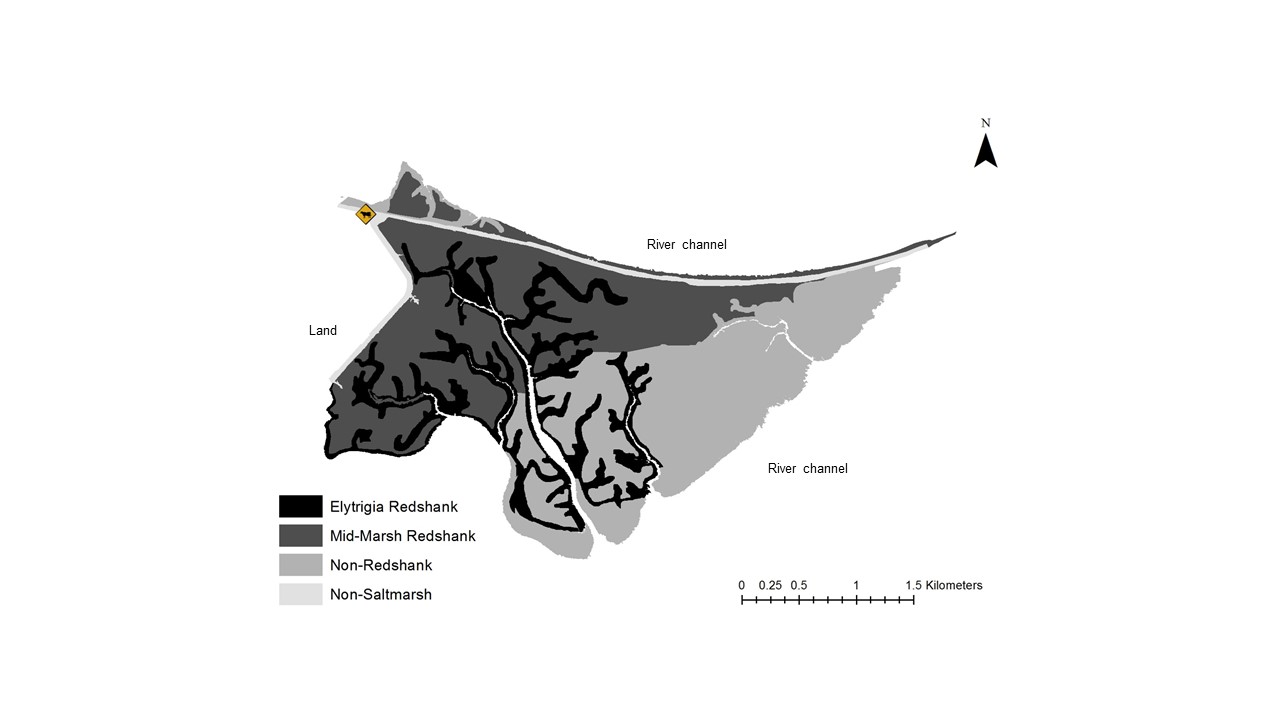


**Supplementary material: Figure 1:** Saltmarsh A. Showing habitat categories and freshwater drinking source (marked with yellow cattle sign).

**
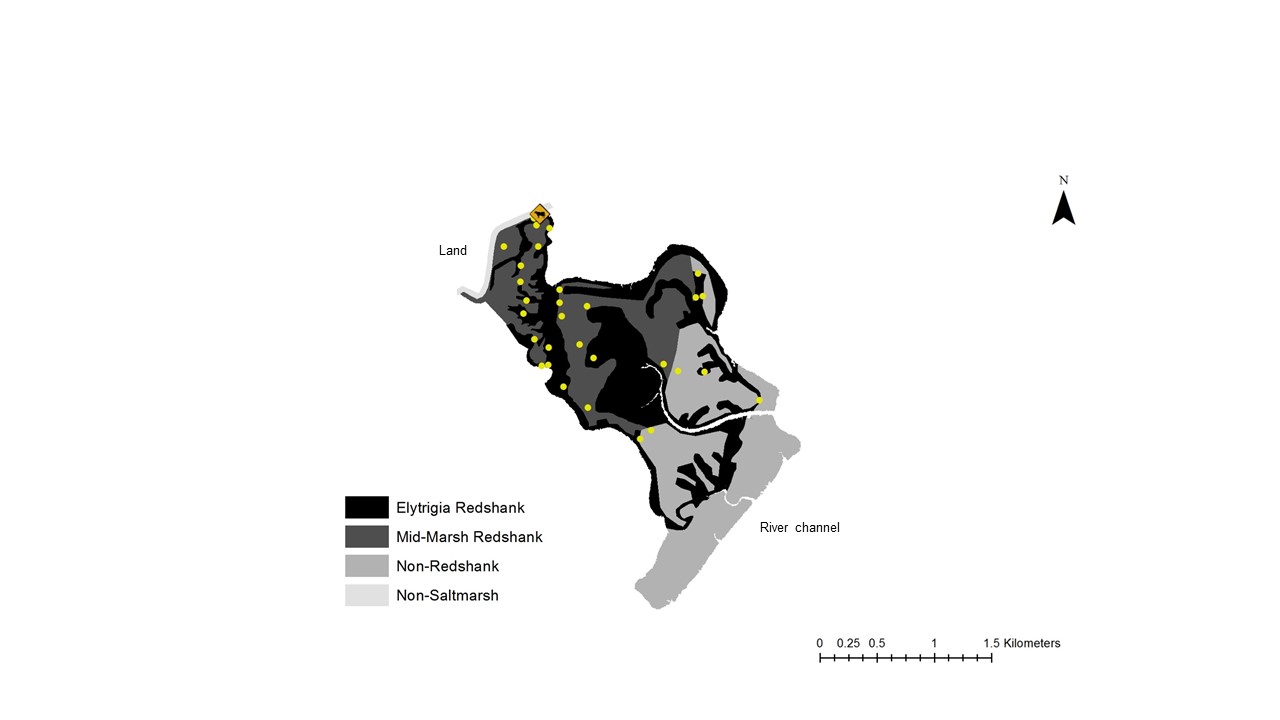
Supplementary material: Figure 2:** Saltmarsh B. Showing habitat categories, freshwater drinking source (marked with yellow cattle sign) and false nest plots (market with a yellow points).


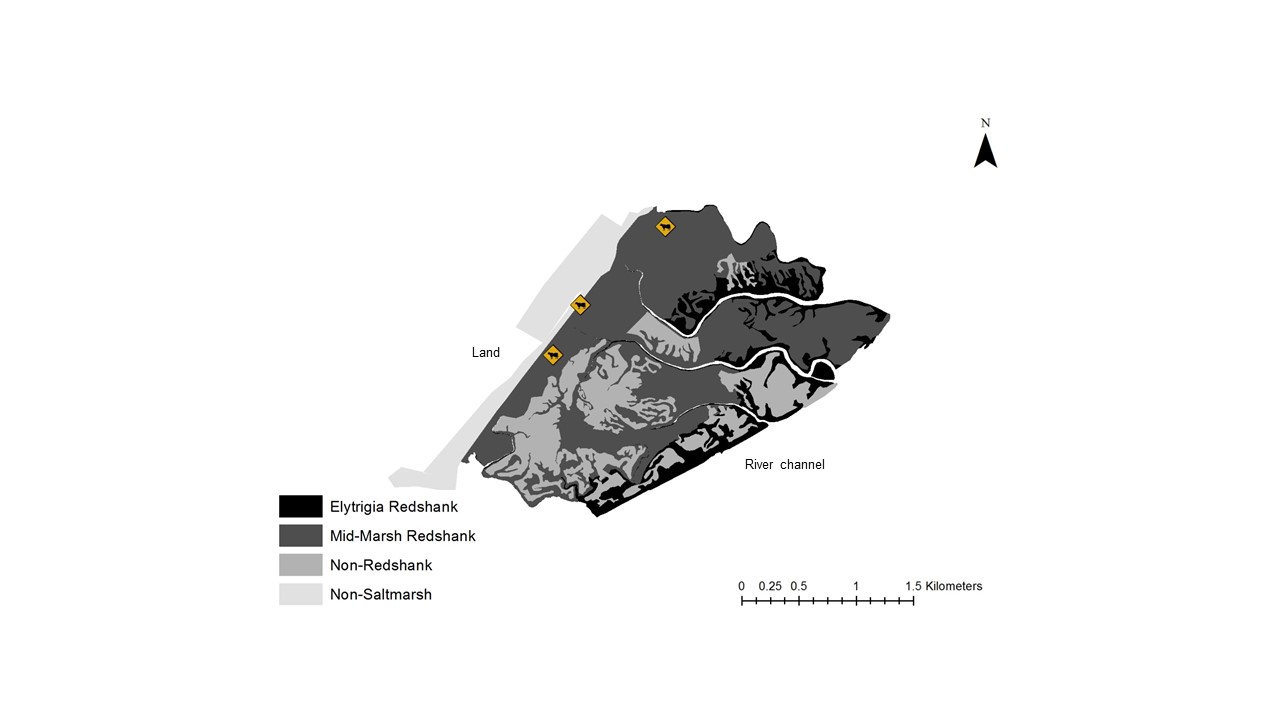


**Supplementary material: Figure 3:** Saltmarsh C. Showing habitat categories and freshwater drinking sources (marked with yellow cattle sign).


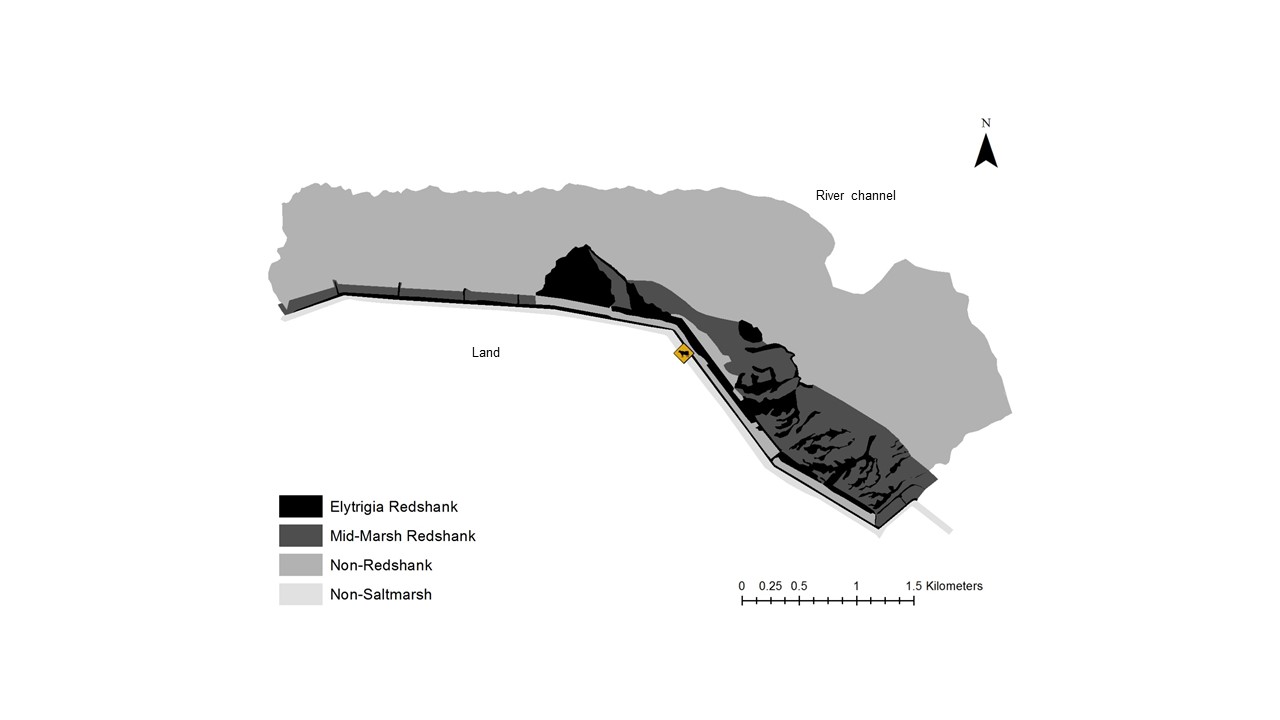


**Supplementary material: Figure 4:** Saltmarsh D. Showing habitat categories and freshwater drinking source (marked with yellow cattle sign).


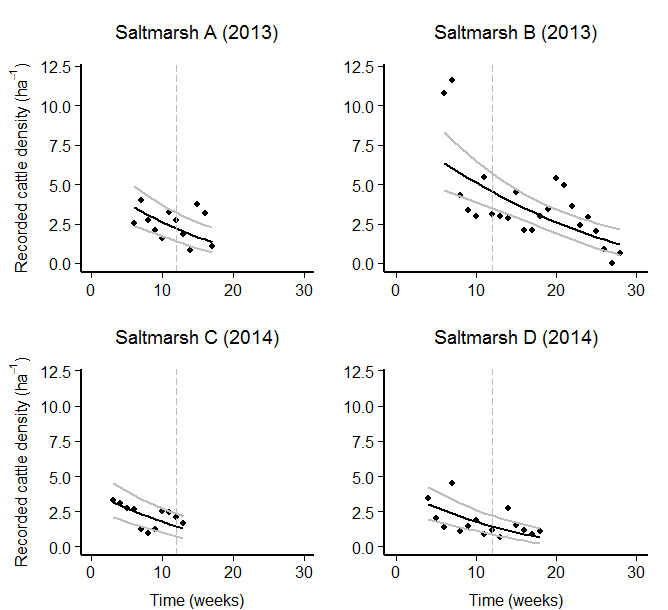


**Supplementary material: Figure 5:** Cattle activity in the non-saltmarsh zone over time. Black lines are back-transformed model-fitted values. Confidence intervals (95%) are indicated by grey lines. The dashed grey vertical lines indicate the end of the redshank nesting season (1^st^ July). Week 1 was the week beginning 14^th^ April. Week 28 (the last week) ended on the 26^th^ October.

**
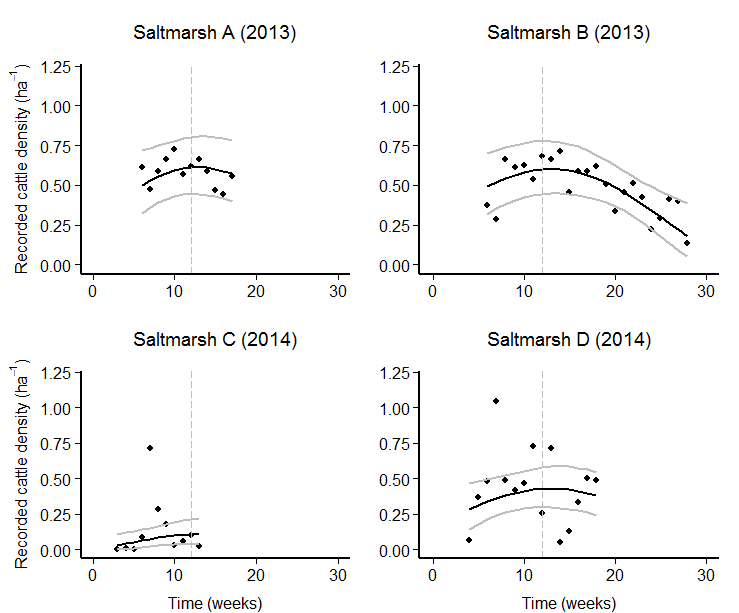
**

**Supplementary material: Figure 6:** Cattle activity in the mid-marsh redshank zone and time. Black lines are back-transformed model-fitted values. Confidence intervals (95%) are indicated by grey lines. The dashed grey vertical lines indicate the end of the redshank nesting season (1^st^ July).


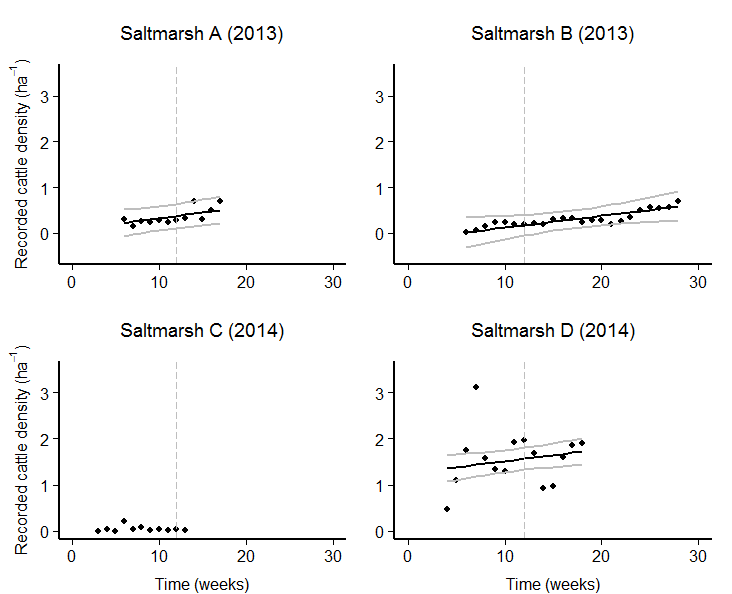


**Supplementary material: Figure 7:** Cattle activity in the *Elytrigia redshank zone* and time. Black lines are back-transformed model-fitted values. Confidence intervals (95%) are indicated by grey lines. The dashed grey vertical lines indicate the end of the redshank nesting season (1^st^ July). Week 1 was the week beginning 14^th^ April. Week 28 (the last week) ended on the 26^th^ October.


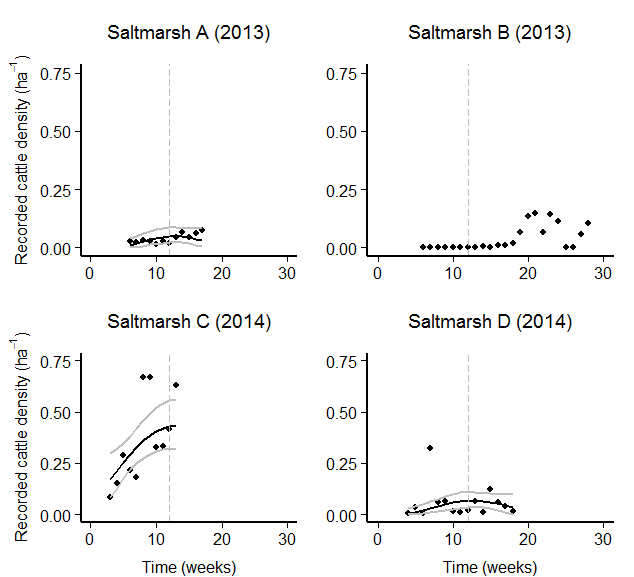


**Supplementary material: Figure 8:** Cattle activity in the non-redshank habitat and time. Black lines are back-transformed model-fitted values. Confidence intervals (95%) are indicated by grey lines. The dashed grey vertical lines indicate the end of the redshank nesting season (1^st^ July). Week 1 was the week beginning 14^th^ April. Week 28 (the last week) ended on the 26^th^ October.
